# Supplementary figures and images for: A new method for long-read sequencing of animal mitochondrial genomes: application to the identification of equine mitochondrial DNA variants
Source: BMC Genomics. 2020 Nov 11;21:785. doi: 10.1186/s12864-020-07183-9 (PMC7661214; doi:10.1186/s12864-020-07183-9)

Quantity of DNA after Repli-G

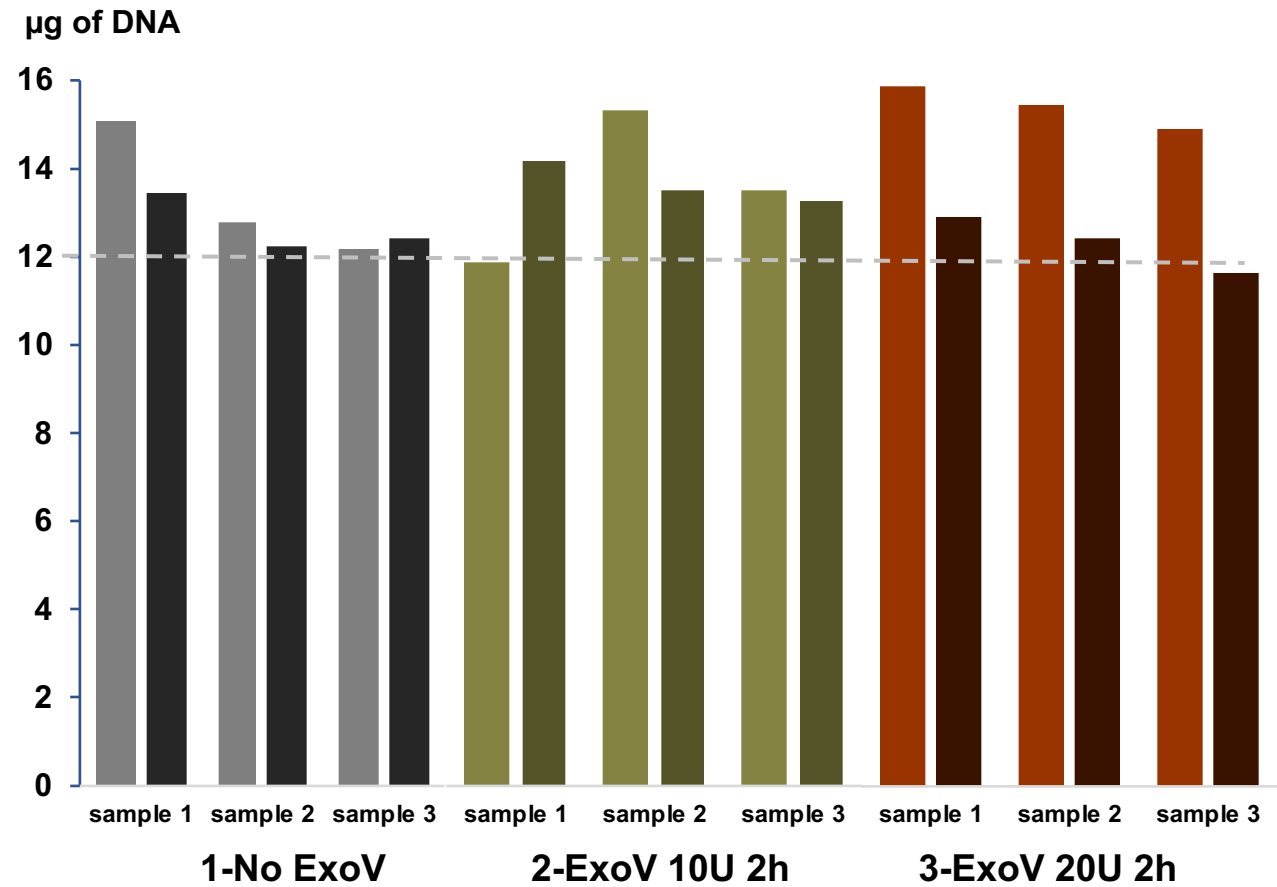

Fragment size after Repli-G

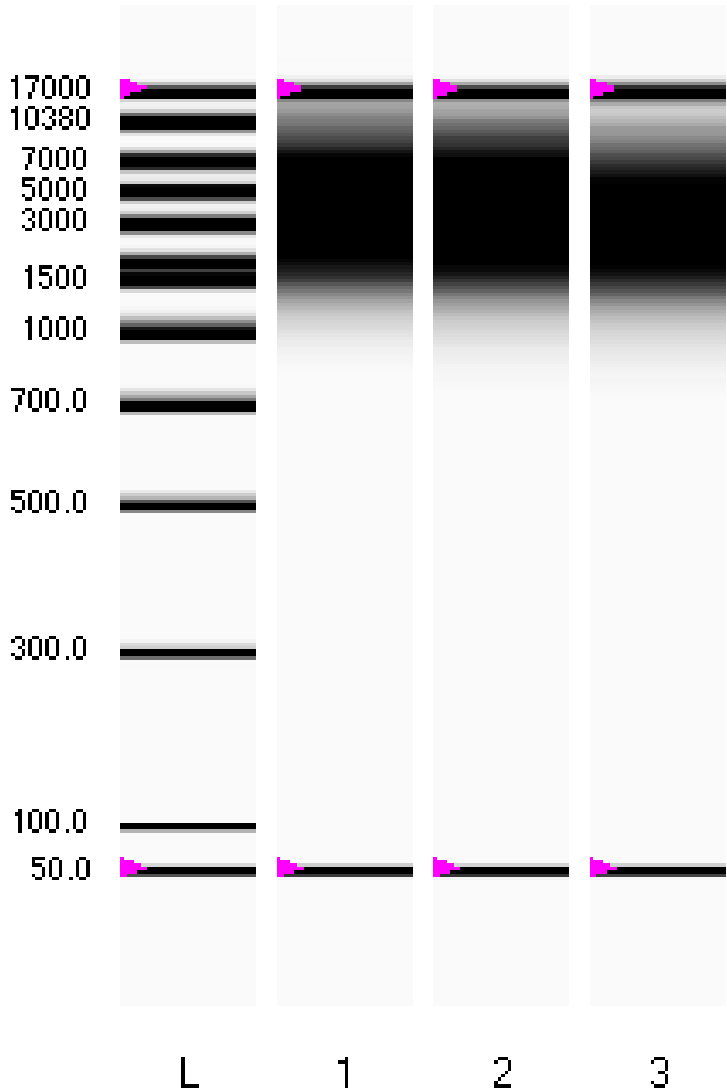

Supplement: Supplementary file 4 — Additional file 4: SI_Figure 1. DNA quantity and fragment size after REPLI-g. [file 12864_2020_7183_MOESM4_ESM.pdf]

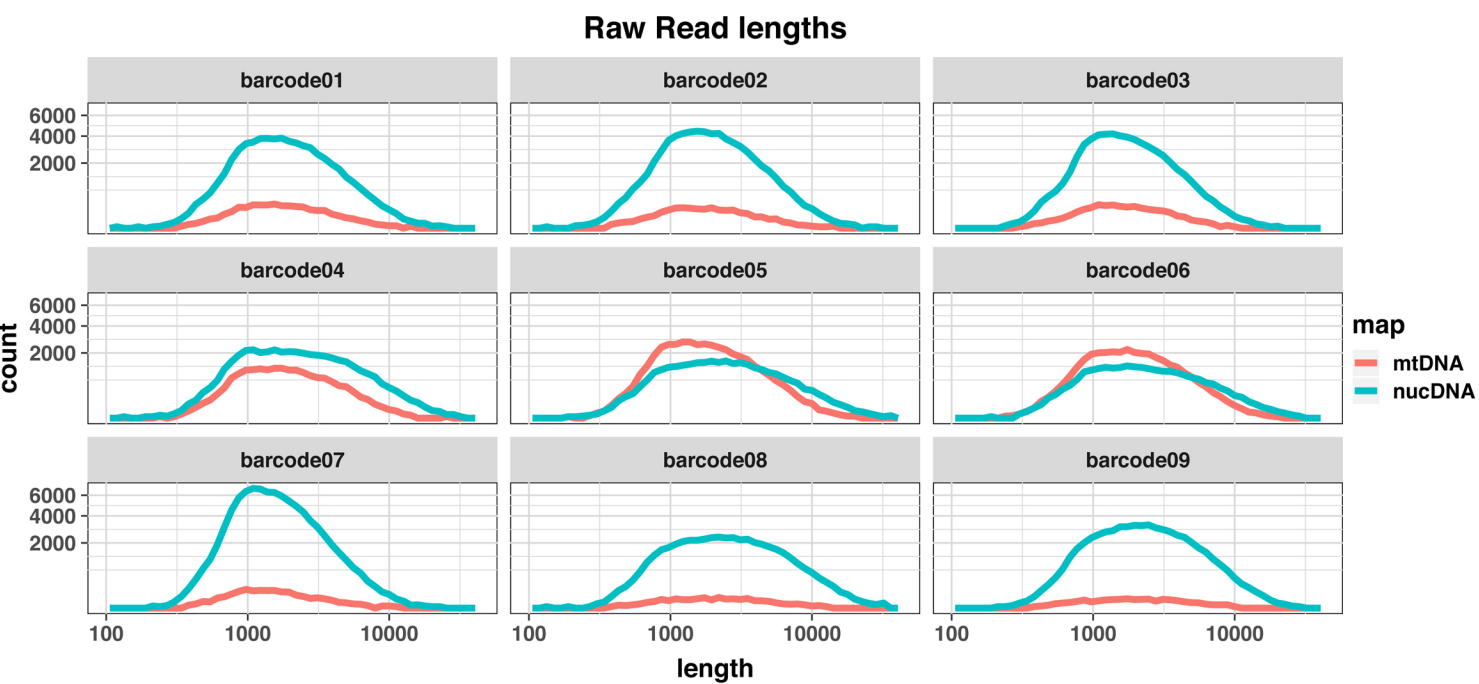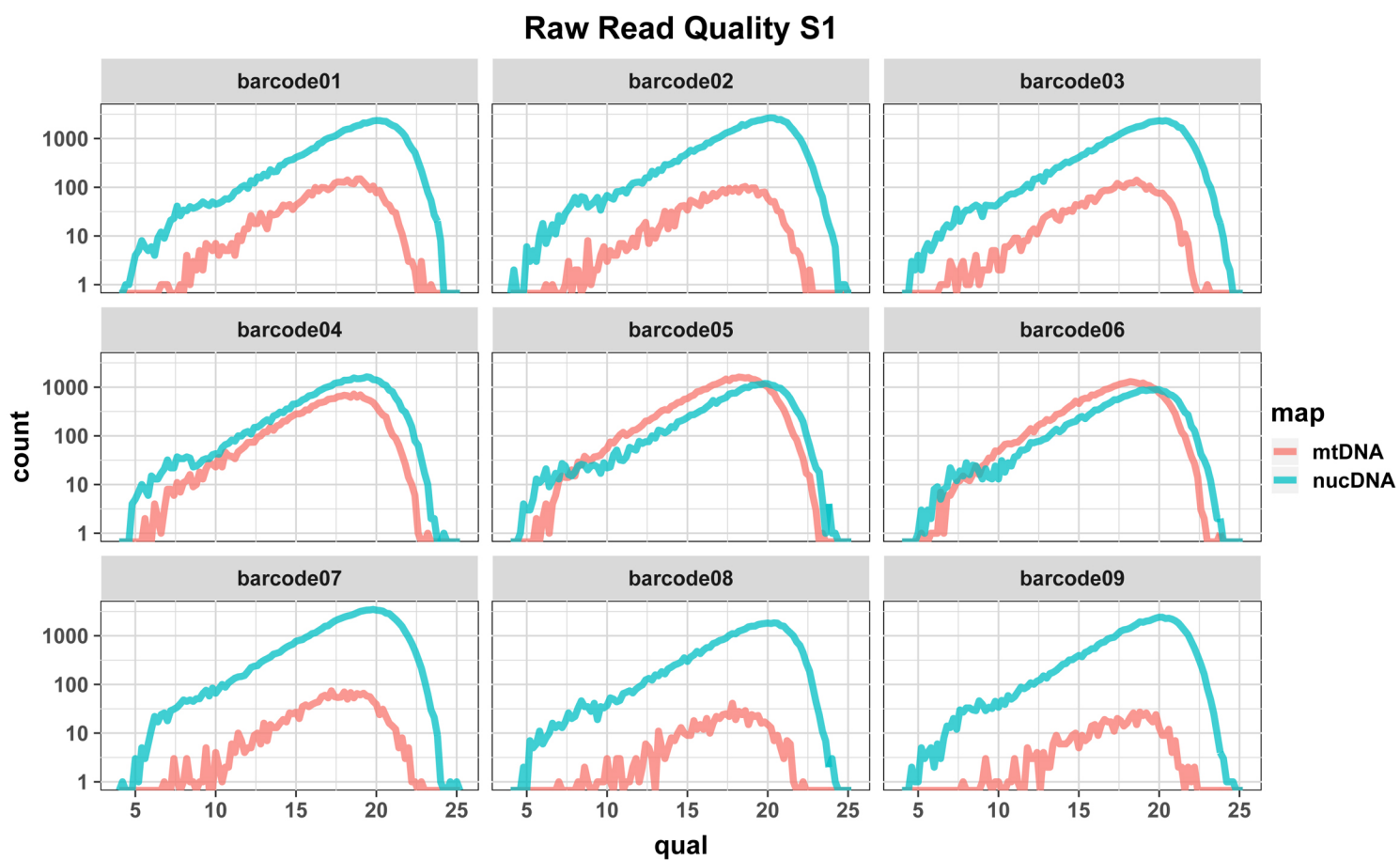

**SI Figure 2**

Supplement: Supplementary file 5 — Additional file 5: SI_Figure 2. Distribution of raw read length and quality by barcode. [file 12864_2020_7183_MOESM5_ESM.pdf]

a. Proportions of mtDNA and nuDNA sequences

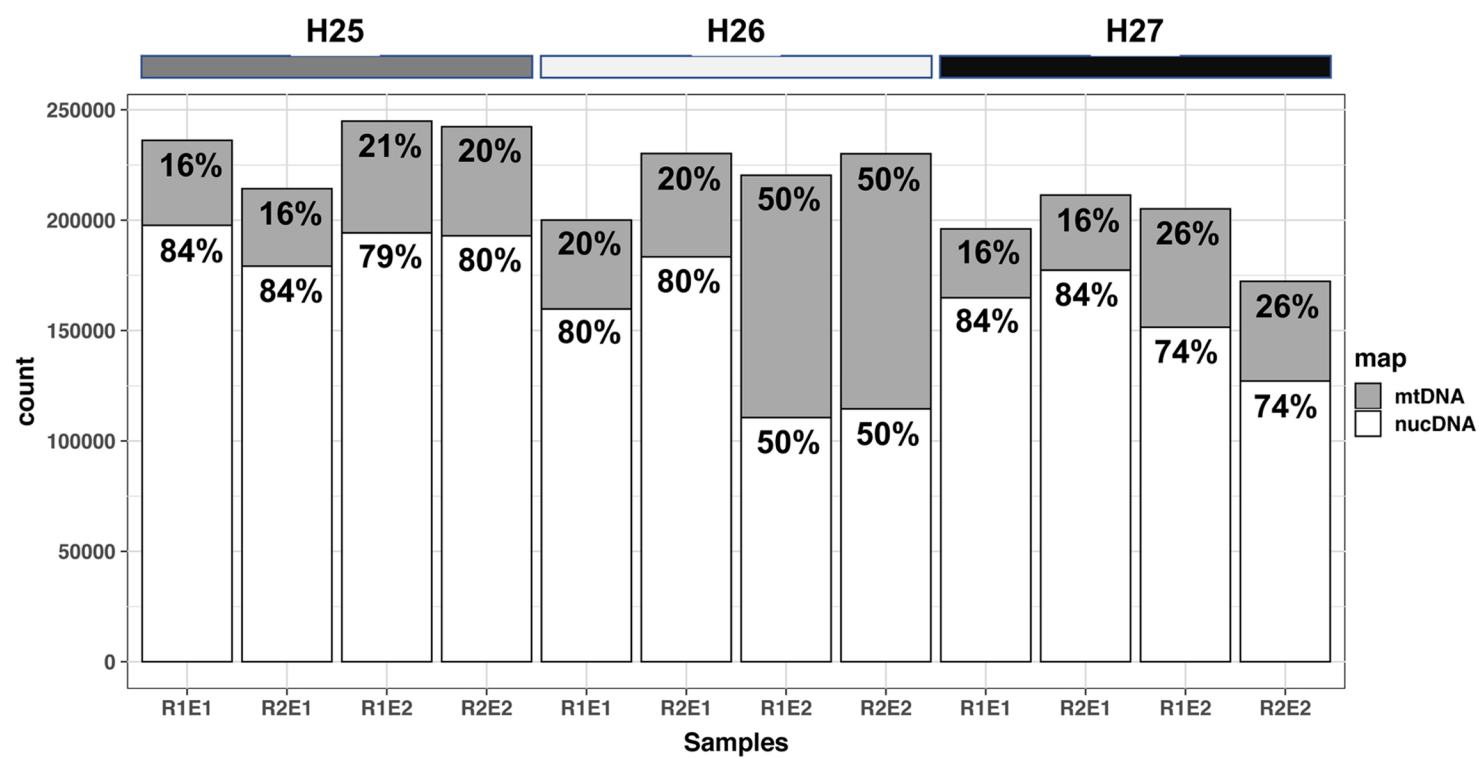

b. Sequence coverage

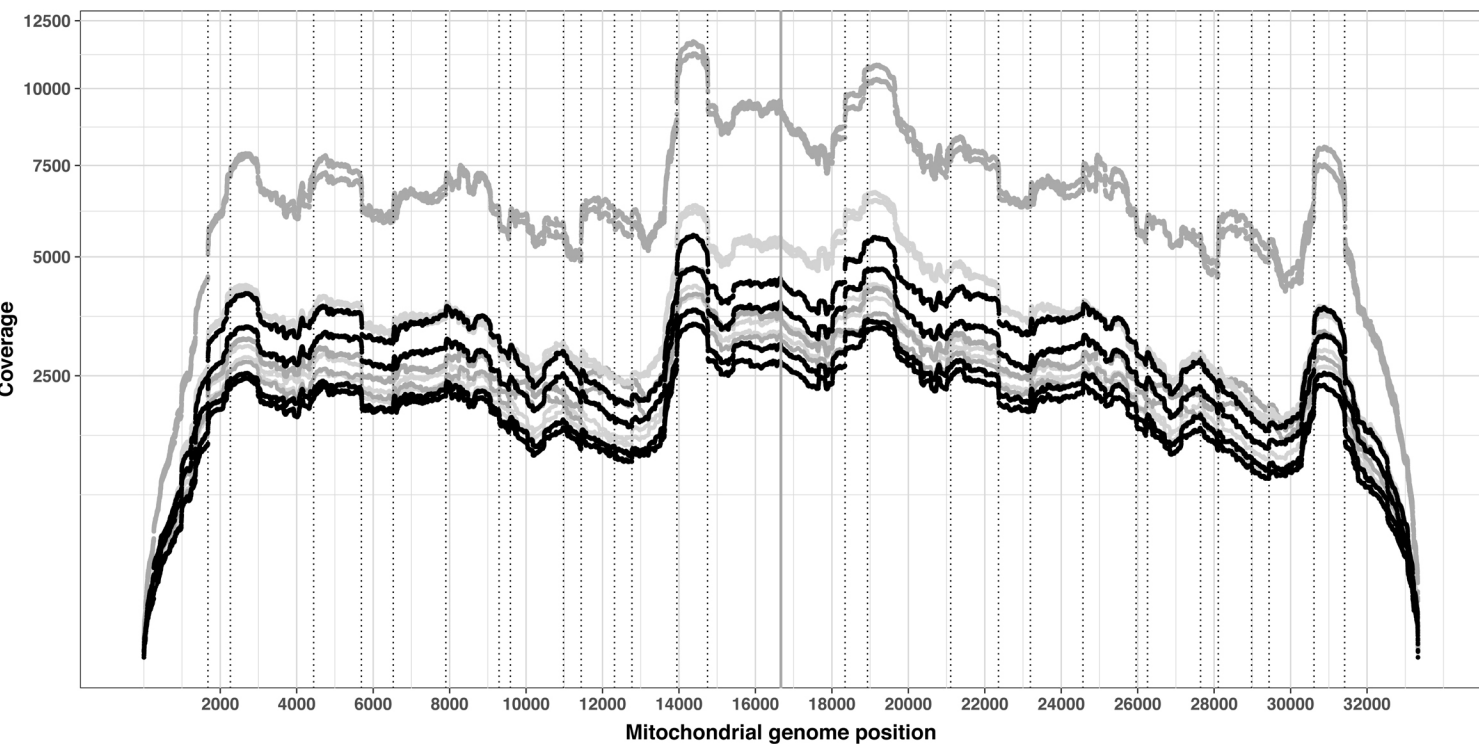

SI Figure 3

Supplement: Supplementary file 6 — Additional file 6: SI_Figure 3. Proportions of mtDNA and nuDNA sequences and sequence coverage in a second sequencing run. [file 12864_2020_7183_MOESM6_ESM.pdf]

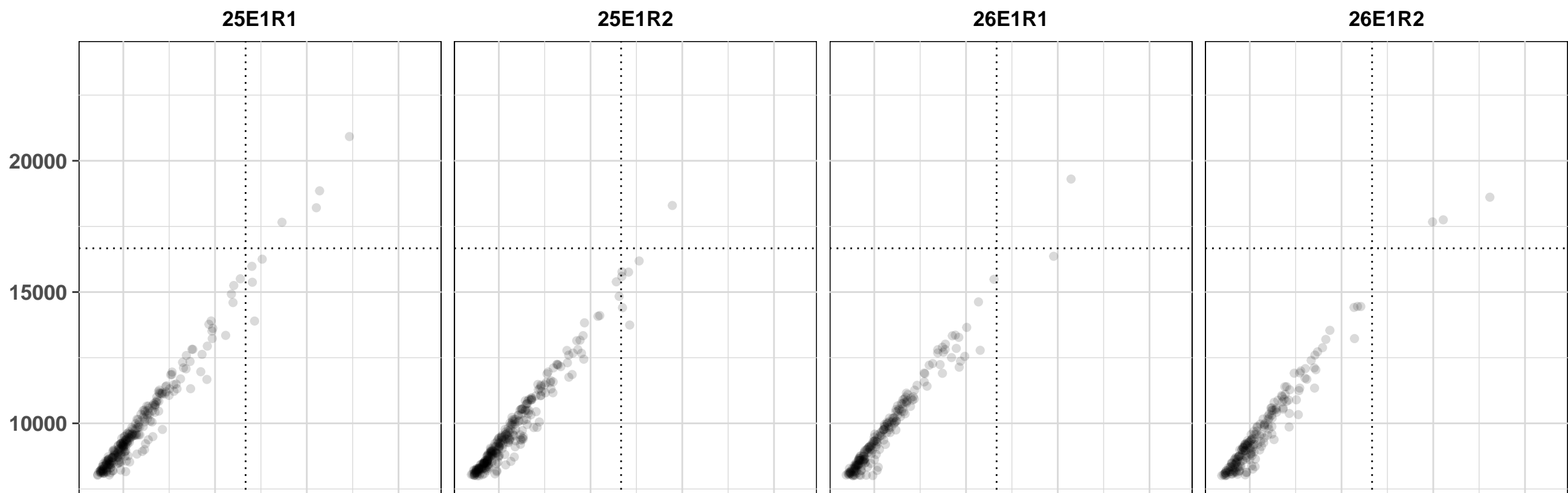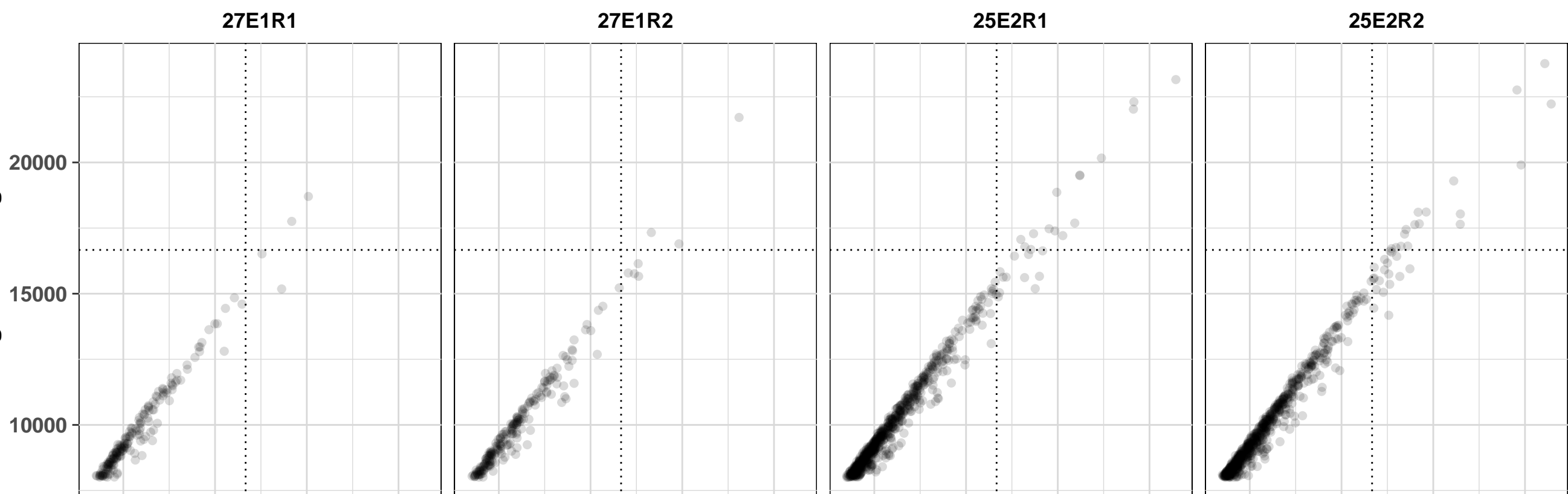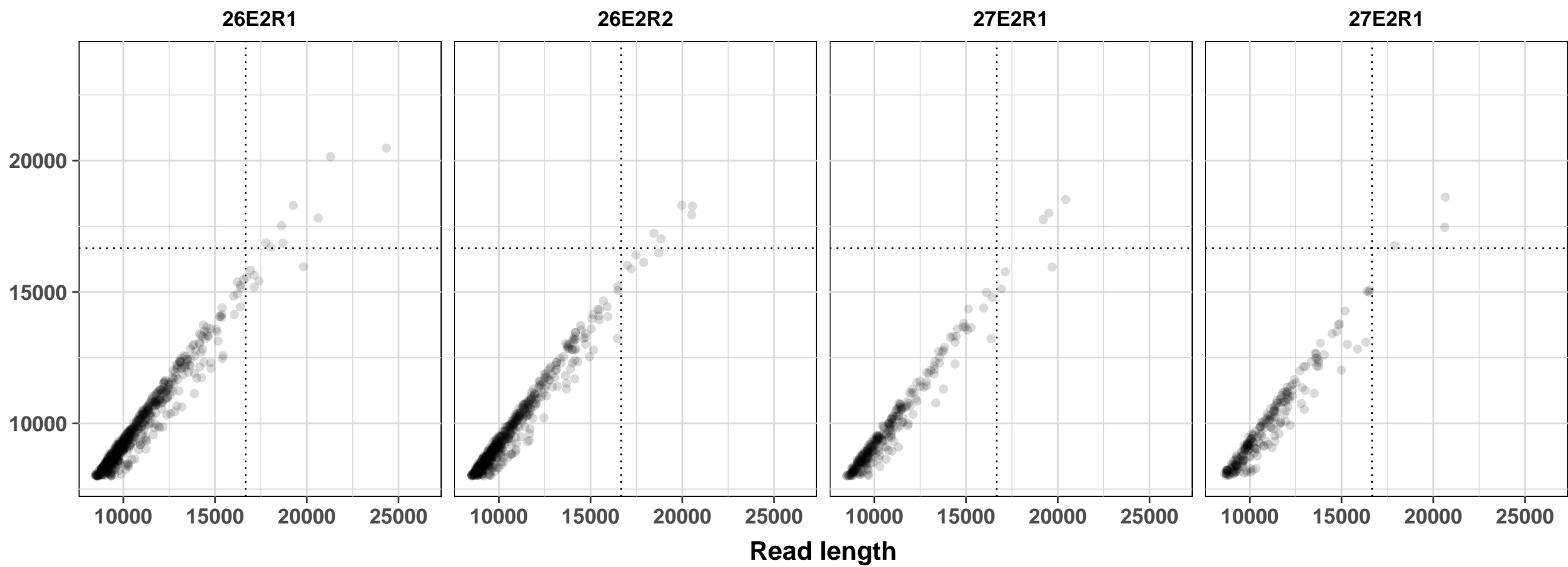

Supplement: Supplementary file 7 — Additional file 7: SI_Figure 4. Distribution of long reads in a second sequencing run. [file 12864_2020_7183_MOESM7_ESM.pdf]

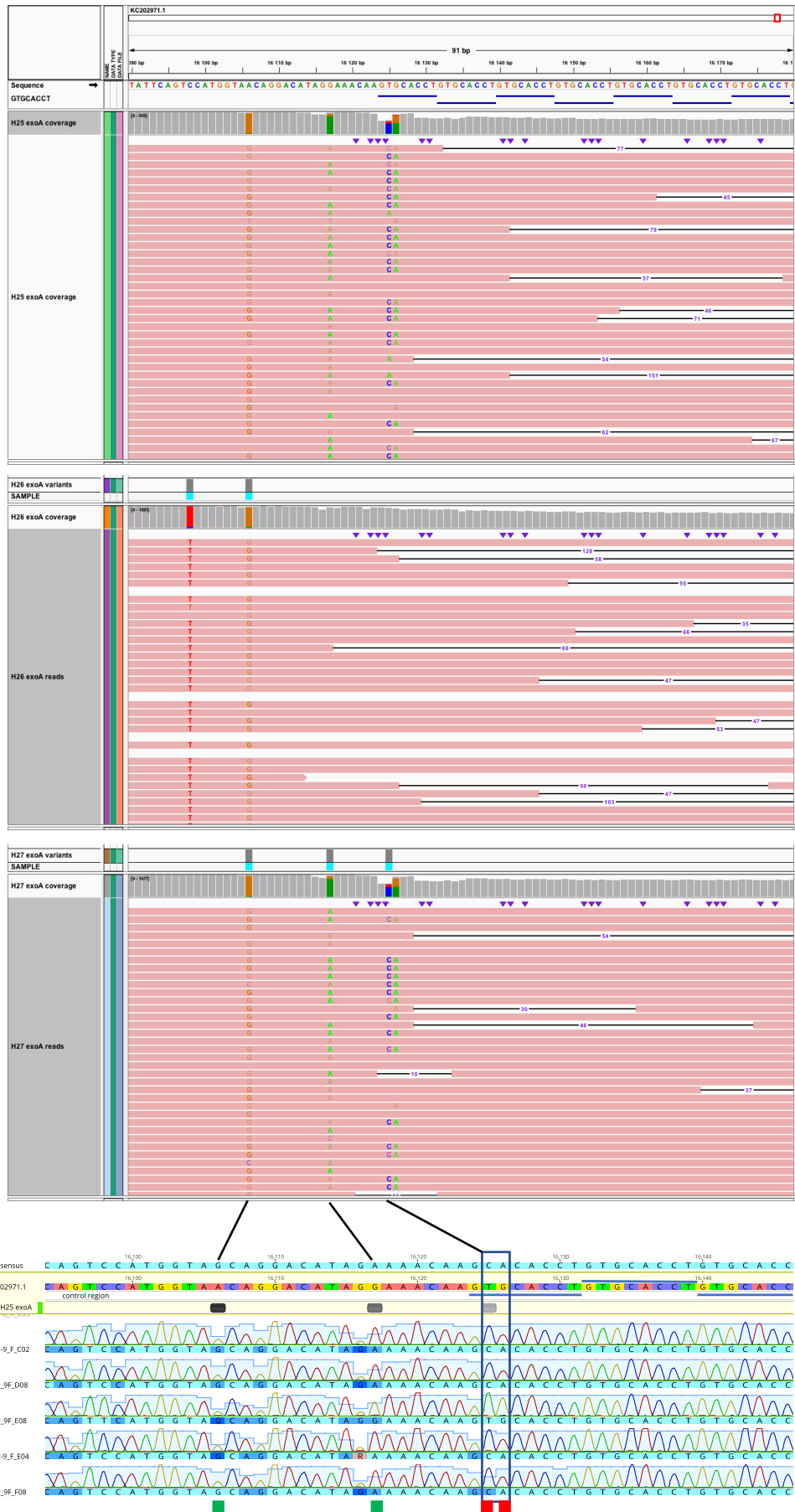

SI Figure 5

Supplement: Supplementary file 8 — Additional file 8: SI_Figure 5. Comparison of long-read and Sanger sequencing in the control region. [file 12864_2020_7183_MOESM8_ESM.pdf]
